# Clinical study of a new skin antiseptic olanexidine gluconate in gastrointestinal cancer surgery

**DOI:** 10.1186/s12893-022-01641-9

**Published:** 2022-05-19

**Authors:** Naoki Kubo, Norihiko Furusawa, Daisuke Takeuchi, Shinichiro Imai, Hitoshi Masuo, Kentaro Umemura, Masaru Terada

**Affiliations:** Department of Surgery, Nagano Prefectural Shinshu Medical Center, 1337, Suzaka, Nagano 382-0091 Japan

**Keywords:** Olanexidine gluconate, Gastrointestinal cancer, Surgical site infection

## Abstract

**Background:**

Surgical site infection (SSI) is a common complication of gastrointestinal surgery. Olanexidine gluconate (OLG) is a novel skin antiseptic that is effective against a wide range of bacteria. The purpose of this study was to evaluate the bactericidal efficacy of OLG in gastrointestinal cancer surgery.

**Methods:**

This retrospective study included a total of 281 patients who underwent gastrointestinal cancer surgery (stomach or colon). The patients were divided into two groups: 223 patients were treated with OLG (OLG group), and 58 patients were treated with povidone-iodine (PVP-I) (control group). The efficacy and safety outcomes were measured as the rate of SSI within 30 days after surgery. In addition, we conducted subgroup analyses according to the surgical approach (open or laparoscopic) or primary lesion (stomach or colon).

**Results:**

There was a significant difference in the rate of SSI between the control group and OLG group (10.3% vs. 2.7%; p = 0.02). There was a significant difference in the SSI rate in terms of superficial infection (8.6% vs. 2.2%; p = 0.0345) but not in deep infection (1.7% vs. 0.5%; p = 0.371). There was no significant difference between the control group and OLG group in the overall rate of adverse skin reactions (5.2% vs. 1.8%; p = 0.157).

**Conclusion:**

This retrospective study demonstrates that OLG is more effective than PVP-I in preventing SSI during gastrointestinal cancer surgery.

## Background

Surgical site infection (SSI) is a postoperative complication of gastrointestinal cancer surgery that causes pain and psychological stress in the patient, prolongs hospital stay and increases medical costs. A high infection rate of 11.3–15.5% has been reported after gastrectomy or colorectal surgery [1]. Several initiatives are aimed at reducing the risk of SSIs [2–4].

The skin is a major source of pathogens that cause SSIs. Therefore, preoperative skin antisepsis has the potential to decrease the risk of SSI [5]. Antiseptics prevent infection by decreasing the number of microorganisms, thereby decreasing the transmission of pathogens. Currently, povidone-iodine (PVP-I) and chlorhexidine gluconate (CHG) are widely used to disinfect surgical sites [6–9]. However, PVP-I may not function well in the presence of organic materials, such as blood or pus, which can rapidly neutralize its bactericidal activity [10], and CHG also does not have sufficient activity to eradicate some pathogens, such as methicillin-resistant *Staphylococcus aureus* (MRSA) and vancomycin-resistant enterococci (VRE) [11].

Olanexidine gluconate (OLG), a novel biguanide antiseptic agent, was introduced in 2015 in Japan for use as a skin disinfectant for surgical sites [12]. OLG exerts strong and fast-acting bactericidal activity against a wide range of bacteria [10]. In both in vitro and in vivo models, the efficacy against MRSA and VRE was higher for OLG than CHG and PVP-I [13], and OLG has a broad spectrum of antibacterial activity against a variety of bacterial strains, including clinical isolates [10]. At present, few reports have explored whether OLG reduces the risk of SSIs after surgery. We retrospectively studied the efficacy of OLG in the surgical treatment of gastrointestinal cancer.

## Materials and methods

### Study group

While PVP-I (Meiji Seika Pharma Co., Ltd., Tokyo, Japan) was previously used to disinfect surgical sites at our institution, OLG (Otuka Pharmaceutical Factory, In, Tokushima, Japan) was adopted for use in April 2016. Preoperative antiseptic use was completely changed from PVP-I to OLG at that time. Patients were assigned to preoperative skin antisepsis with OLG or PVP-I to evaluate the comparative effectiveness of the two preoperative skin preparations for the prevention of SSIs after gastrointestinal cancer surgery. The medical records of patients who underwent surgery for primary gastric or colon cancer between April 2015 and May 2020 were retrospectively reviewed. The method of wound closure was the same between both groups.

**Fig. 1:**
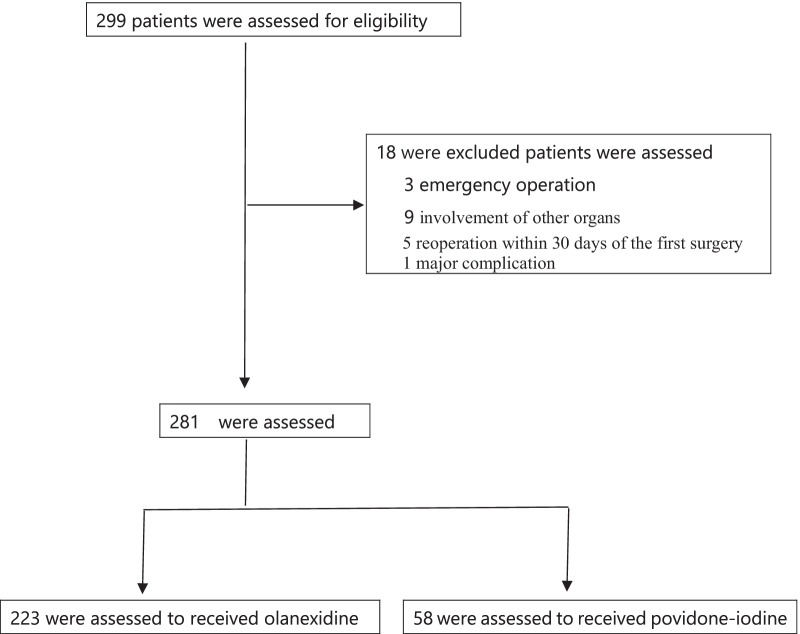
CONSORT for the trial

A total of 299 patients diagnosed with primary gastric or colon cancer underwent gastrectomy or colectomy combined with lymphadenectomy. The exclusion criteria were emergency operations, involvement of other organs, and reoperation within 30 days of the first surgery. A total of 18 patients were excluded, and 281 patients were finally evaluated prospectively. Among the patients who met the inclusion criteria between April 2015 and May 2020, 58 patients who underwent conventional skin disinfection with PVP-I and 223 patients who underwent conventional skin disinfection with OLG were divided into the control group and OLG group, respectively (Fig. 1).

PVP-I was applied by wiping down the skin surface with gauze soaked with the drug, and OLG was applied using a sterile prepacked applicator. All patients received antibiotic prophylaxis during and after surgery, but not preoperative oral antibiotics. All patients underwent mechanical bowel preparation and were treated using a wound protector (Alexis wound protector, Applied Medical, Rancho Santa Margarita, CA, USA) during the operation.

We investigated the correlations between preoperative skin disinfection and the incidence of SSI, and estimated the risk factors for SSI.

### Trial outcome

The efficacy outcome was superficial or deep SSI within 30 days after the operation, according to the National Healthcare Safety Network definitions of the Centers for Disease Control and Prevention (CDC) [5]. All patients were checked daily for signs of infection during admission. After discharge, all patients had outpatient visits within 30 days after surgery to check for signs of infection. They were also expected to visit an outpatient or emergency department immediately whenever there were signs of infection.

Skin or subcutaneous and deep tissue infections in purulent drainage, cultured organisms, procedural intervention due to pain, swelling, erythema, fever, and the diagnosis made by the surgeon were generally considered SSIs. Safety outcomes were defined as the rate of adverse skin reactions, such as skin irritation, erythema or pruritus, in the area of application of the disinfectant. We reviewed the patient records and collected data on patient sex, age, body mass index (BMI), operation time, amount of bleeding during the operation, comorbidities, approach (open or laparoscopy), site, tumor size, stage, postoperative complications, and postoperative length of hospital stay [14]. We conducted a subgroup analysis of the primary outcome in subgroups defined according to the surgical approach (laparotomy or laparoscopic) and the site of the primary lesion (stomach or colon). This retrospective study was designed and independently performed with approval from the ethics committee of Nagano Prefectural Shinshu Medical Center in accordance with the principles of the Declaration of Helsinki.

### Statistical analysis

Statistical analyses were conducted using EZR (Saitama Medical Center, Jichi Medical University), which is a graphical user interface for R (The R Foundation for Statistical Computing, version 3.4.1). Associations among patient characteristics, the antiseptics and SSI were evaluated using the Chi-square test and Student’s t test. Multivariate analysis was also performed using a logistic regression model to assess factors that predicted SSI development. Two-sided P values lower than 0.05 were considered to indicate statistical significance.

## Results

### Patient background

The characteristics of the patients and preoperative skin antisepsis are shown in Table 1. There were no significant differences in baseline patient characteristics between the two groups with regard to age, sex, BMI, diabetes mellitus, preoperative albumin level, respiratory disease, anticoagulant, primary site (stomach or colon), American Society of Anesthesiologists (ASA) grade, operation time, perioperative blood loss, transfusion, stage, complications other than SSIs, or adverse skin reactions (Table 1).

**Table 1:** Patient and operative characteristics

Patient characteristics	OLG group (n = 223)	Control group (n = 58)	P value
Mean age (range)	73.2 ± 10.7	73.9 ± 10.2	0.853
Gender			0.455
M	133 (59.6%)	31 (53.4%)	
F	90 (40.4%)	27 (46.6%)	
Mean BMI ± SD	22.1 ± 3.5	22.4 ± 3.7	0.6
Diabetes mellitus (%)	66 (29.6%)	19 (32.8%)	0.634
Alb	3.89 ± 0.52	3.86 ± 0.57	0.752
Respiratory disease	39 (17.5%)	8 (13.8%)	0.56
Anticoagulant	41 (16.7%)	10 (17.2%)	1
Primary lesion			1
Stomach	66 (29.6%)	17 (29.3%)	
Colon	157 (70.4%)	41 (70.7%)	
ASA			0.495
1.2	171 (76.7%)	42 (72.4%)	
3	52 (23.3%)	16 (27.6%)	
Mean operation time ± SD	303.0 ± 108.8	297.2 ± 134.9	0.732
Bleeding (ml)	130.4 ± 244.5	133.3 ± 152.8	0.932
Approach			0.000171
Open	91 (43.8%)	42 (72.4%)	
Laparoscopy	117 (56.2%)	16 (27.6%)	
Transfusion	5 (2.3%)	1 (1.7%)	1
Stage			0.641
0, I, II	150 (67.3%)	37 (63.8%)	
III, IV	73 (32.7%)	21 (36.2%)	
Leakage	4 (1.8%)	5 (8.6%)	0.0205
Complication except SSI	51 (22.9%)	18 (31.0%)	0.232
Postoperative length of hospital stay (days)	15.8 ± 10.4	20.0 ± 15.8	0.0136
Adverse skin reaction (all)	4 (1.8%)	3 (5.2%)	0.157
Skin irritation	2 (0.9%)	0 (0%)	1
Erythema	3 (1.3%)	3 (5.2%)	0.105
Pruritus	1 (0.4%)	1 (1.7%)	0.371

Control group: PVP-I group

However, there were significant differences between the control and OLG groups in terms of approach (laparotomy/laparoscopy: 42/16 vs. 91/117, p = 0.000171), leakage (yes/no: 4/219 vs. 5/53, p = 0.0205) and postoperative length of hospital stay (15.8 vs. 20.0 days, p = 0.0136).

### Surgical site infection

The overall incidence of SSI was 4.3% (n = 12). Six patients in the control group (10.3%) and 6 in the OLG group (2.7%) developed SSIs (Table 2), and a significant difference was observed between the two groups (p = 0.02). In the control and OLG groups, the rates of superficial infection were 8.6% and 2.2%, respectively (p = 0.0345), and the rates of deep infection were 1.7% and 0.5%, respectively (p = 0.371).

**Table 2:** Effect of surgical site infection

All	OLG group (n = 223)	Control group (n = 58)	P value
Surgical site infection	6 (2.7%)	6 (10.3%)	0.02
Superficial incisional	5 (2.2%)	5 (8.6%)	0.0345
Deep incisional	1 (0.5%)	1 (1.7%)	0.371

Open	OLG group (n = 91)	Control group (n = 42)	
Surgical site infection	2 (2.2%)	4 (9.5%)	0.0789
Superficial incisional	2 (2.2%)	4 (9.5%)	0.0789
Deep incisional	0 (0%)	0 (0%)	

Laparoscopy	OLG group (n = 132)	Control group (n = 16)	
Surgical site infection	4 (3.0%)	2 (12.5%)	0.127
Superficial incisional	3 (2.3%)	1 (6.3%)	0.37
Deep incisional	1 (0.8%)	1 (6.3%)	0.205

Stomach	OLG group (n = 66)	Control group (n = 17)	
Surgical site infection	0 (0%)	1 (5.9%)	0.205
Superficial incisional	0 (0%)	1 (5.9%)	0.205
Deep incisional	0 (0%)	0 (0%)	

Colon	OLG group (n = 157)	Control group (n = 41)	
Surgical site infection	6 (3.8%)	5 (12.2%)	0.0523
Superficial incisional	5 (3.2%)	4 (9.8%)	0.0904
Deep incisional	1 (0.6%)	1 (2.4%)	0.372

Control group: PVP-I group

In the subgroup analysis, the incidence of SSI was 4.5% for laparotomy and 4.1% for laparoscopy. However, among patients treated with laparotomy, 4 in the control group (9.5%) and 2 in the OLG group (2.2%) developed an SSI, and there was no significant difference between the two groups (p = 0.0789). Similarly, among patients treated with laparoscopy, 2 in the control group (12.5%) and 4 in the OLG group (3.0%) developed an SSI, but there was no significant difference between the two groups (p = 0.127) (Table 2). Regarding the primary site lesion, the incidence of SSI was 1.2% for the stomach and 5.6% for the colon. However, among patients who underwent gastrectomy, 1 in the control group (5.9%) and 0 in the OLG group (0%) developed an SSI, and there was no significant difference between the two groups (p = 0.205). Similarly, among patients who underwent colectomy, 5 in the control group (12.2%) and 6 in the OLG group (3.8%) developed an SSIs, but there was no significant difference between the two groups (p = 0.0523) (Table 2).

The factors found to be associated with SSI are shown in Table 3. Diabetes, ASA grade, anticoagulant administration and the use of OLG significantly influenced the incidence of SSI. A significantly higher incidence of anastomotic leakage in the control group did not affect the development of SSI. The rates of OLG use in patients with and without SSI were 50.0% and 90.4%, respectively (p = 0.02). The risk factors that tended to be correlated with the development of SSIs (p < 0.05) in univariate analyses were mainly included in a multivariate analysis. Multivariate analysis also demonstrated that the use of OLG was the only significant risk factor for the development of SSIs (OR 0.142, 95% CI 0.0332–0.610, p = 0.00862) (Table 4).

**Table 3:** Patient characteristics and the incidence of SSI

Patient characteristics	SSI− (n = 269)	SSI+ (n = 12)	P value
Mean age (range)	72.9 ± 10.6	77.4 ± 11.3	0.151
Gender			0.37
M	155 (57.6%)	9 (75.0%)	
F	114 (42.4%)	3 (25.0%)	
Mean BMI ± SD	22.2 ± 3.5	22.9 ± 3.9	0.485
Diabetes mellitus (%)	78 (29.0%)	7 (58.3%)	0.0489
Albumin ± SD	3.89 ± 0.53	3.71 ± 0.52	0.244
Respiratory disease	43 (16.0%)	4 (33.3%)	0.122
Anticoagulant	28 (17.1%)	5 (45.5%)	0.032
ASA			0.0434
1.2	207 (77.0%)	6 (50.0%)	
3	62 (23.0%)	6 (50.0%)	
Mean operation time ± SD	300.1 ± 110.0	339.7 ± 191.8	0.242
Bleeding (ml)	128.4 ± 222.6	188.6 ± 340.0	0.373
Approach			1
Open	127 (50.0%)	6 (50.0%)	
Laparoscopy	127 (50.0%)	6 (50.0%)	
Transfusion	6 (2.2%)	0 (0%)	1
Use of olanexidine	217 (90.4%)	6 (50.0%)	0.02
Primary lesion			0.118
Stomach	82 (34.2%)	1 (8.3%)	
Colon	187 (65.8%)	11 (91.7%)	
Tumor size (cm)	4.65 ± 2.56	4.25 ± 2.13	0.595
Stage			0.543
0, I, II	180 (66.9%)	7 (58.3%)	
III, IV	89 (33.1%)	5 (41.7%)	
Leakage	9 (100%)	0 (0%)	1
Complication except SSI	63 (23.5%)	6 (50.0%)	0.0782
Adverse skin reaction (all)	7 (2.6%)	0 (0%)	1

**Table 4:** Multivariate analysis of risk factor s for developing SSI

Factor	Effect size (95% CI)	P value
Age	1.09 (0.982–1.200)	0.107
Male gender	2.04 (0.418–9.990)	0.378
Diabetes mellitus	3.67 (0.908–14.900)	0.068
Anticoagulant	1.44 (0.310–6.690)	0.642
ASA (1.2 or 3)	1.87 (0.413–8.440)	0.417
Site (stomach or colon)	0.217 (0.0200–2.360)	0.209
Approach (open or laparoscopy)	0.636 (0.138–2.930)	0.562
Use of olanexidine	0.160 (0.0365–0.700)	0.0150
Complication except SSI	2.60 (0.657–10.300)	0.173

In 7 of the 12 patients with SSI, the culture specimens were positive for bacterial growth. Table 5 summarizes the distribution of organisms isolated from the SSI patients in both groups. The most common organism was *Enterococcus faecalis* in the OLG group and *Streptococcus constellatus* in the control group.

**Table 5:** Organisms isolated from surgical sites (percentage)

Organisms	OLG group (n = 6)	Control group (n = 6)
*Enterococcus faecalis*	2 (33.3%)	0 (0%)
*Enterococcus avium*	1 (16.7%)	0 (0%)
*Enterobacter aerogenes*	1 (16.7%)	0 (0%)
*Enterobacter cloacae*	1 (16.7%)	0 (0%)
*Pseudomonas aeruginosa*	1 (16.7%)	0 (0%)
*Klebsiella pneumoniae*	1 (16.7%)	0 (0%)
*Escherichia coli*	1 (16.7%)	1 (16.7%)
*Streptococcus constellatus*	0 (0%)	2 (33.3%)
MSSA	0 (0%)	1 (16.7%)
*Citrobacter freundii*	0 (0%)	1 (16.7%)
*Corynebacterium* sp	0 (0%)	1 (16.7%)
γ-streptococcus	0 (0%)	1 (16.7%)

MSSA: methicillin-sensitive *Staphylococcus aureus*; Control group: PVP-I group

## Conclusion

In this retrospective analysis, we found that the risk of SSI after gastrointestinal cancer surgery was significantly lower when OLG was used for preoperative skin preparation than when PVP-I was used. The incisional SSI rates were 2.7% in the OLG group and 10.3% in the control group. This result could directly imply the efficacy of olanexidine for surgical skin antisepsis in gastrointestinal surgery.

SSI can occur as a complication after surgery for gastrointestinal cancer and causes pain and psychological stress in the patient, prolongs hospital stays and increases healthcare costs [15]. A high infection rate of 11.3–15.5% has been reported after gastrectomy or colorectal surgery [1]. Several initiatives are aimed at reducing the risk of SSIs [2–4]. Many perioperative measures to reduce SSI have been reported, including enhanced nutritional support, perioperative oxygenation, different surgical techniques, wound dressing and the use of an antimicrobial agent [13].

The skin is a major source of pathogens that cause SSIs. Therefore, preoperative skin antisepsis may reduce the risk of SSI [5]. Antiseptics prevent infection by decreasing the number of microorganisms and thereby reduce the transmission of pathogens [10]. Currently, PVP-I, CHG and other alcohol-based preparations are widely used to disinfect surgical sites. The CDC guidelines recommend that skin preparation be performed with an alcohol-containing agent only if there are no contraindications to its use, while other guidelines do not favor one antiseptic agent over another for skin preparation [16]. PVP-I and CHG both have broad-spectrum antibacterial effectiveness. However, PVP-I may not function well in the presence of organic materials, such as blood or pus, which can rapidly neutralize its bactericidal activity [10]. CHG also does not have sufficient activity to eradicate some pathogens, such as MRSA and VRE [11]. Furthermore, alcohol-based products are highly flammable and can burn the skin if they are not allowed sufficient time to dry [17–19]. Therefore, it is necessary to identify more effective antiseptics for surgical site preparation.

OLG, a novel biguanide antiseptic agent, has been commercially available since 2015 in Japan for use as a skin disinfectant for surgical sites [12]. It disrupts membrane integrity by binding to the cell membrane, resulting in irreversible leakage of intracellular components, which is the mechanism underlying its bactericidal and fungicidal activities [13]. However, few clinical investigations have explored the use of OLG as a preoperative disinfectant in digestive surgery.

While Asukai et al. performed a retrospective study in the field of orthopedics, they found no significant difference between OLG and PVP-I [14]. On the other hand, Obara et al. performed a randomized study in clean contaminated gastrointestinal and hepatobiliary pancreatic surgery and found a significant difference between OLG and PVP-I, which is nearly equivalent to our study [20]. Almost all clean surgeries performed in the orthopedic department were included in this study, and the rate of SSI was low; therefore, it was difficult to identify a difference. However, the risk of SSI is higher in gastrointestinal surgery than in orthopedic surgery, and it is therefore possible to identify a significant difference in this group. Thus, the use of OLG may be more effective in surgeries with a high risk of SSI.

Many factors affect SSI and have been previously reported in digestive surgery. Known risk factors for SSI include ASA grade, operation time, diabetes, BMI, and intraoperative blood transfusion. Laparoscopic surgery is considered to reduce the incidence of SSIs. Other reports include age, sex, use of prophylactic antibiotics, ostomy, preoperative use of nonabsorbable oral antibiotics, smoking, type of skin closure, and total nutrition [21–29]. However, few common risk factors were identified in our surveillance data. This finding suggests that the risk factors for SSI may vary in accordance with the changing conditions experienced during surgery. The widespread use of laparoscopic surgery is a condition that changed markedly during the study period. While laparoscopic surgery is minimally invasive and usually performed with less blood loss than is observed during open surgery, it requires a longer operation time. The advantageous features of laparoscopic surgery may contribute to a decreased risk of SSI, as suggested in a previous study [30]. In our study, although the difference was not significant between laparoscopic surgery and open surgery in the rate of SSI, this might be due to the very low number of laparoscopic surgeries in the control group. Since patients who underwent laparoscopic surgery were mainly included in the OLG group, it is possible that the rate of SSI was significantly lower in the OLG group, and this effect was therefore further examined for each approach in subgroup analysis. The results showed that there was no significant difference, but the rate of SSI was lower in the OLG group than in the control group in both the open and laparoscopic surgery subgroup. Therefore, OLG may reduce SSI regardless of the selected approach (open or laparoscopic).

On the other hand, there was no significant difference between the OLG group and the control group for either gastric cancer or colorectal cancer. However, in the colorectal cancer patients, for whom the rate of SSI was high, while the rate of SSI was originally low in gastric cancer, the rate of SSI was considerably lower in the OLG group. This result also shows that the use of OLG may be more effective in surgery with a high risk of SSI.

Regarding the organisms isolated from the surgical sites, the most common was *E. faecalis* in the OLG group and *S. constellatus* in the control group. The purpose of surgical site skin disinfection is to reduce the skin flora. Most organisms cultured in the OLG group were enteric bacteria that could not be reduced by disinfectant, and few organisms from the epidermis and outside that could be reduced by disinfectant were found. This is considered to be very useful for surgical site skin disinfection.

Our study has several limitations. First, this was a single-center retrospective study across different time periods in which the number of cases was small. It would have been useful to compare data within the same operative method, if possible, but this study was performed using the described methods for primary gastric or colorectal cancer since the number of cases is small in this mid-sized general hospital in Japan. The content was nearly uniform since the operative procedure and perioperative management used during surgery and the preoperative and postoperative periods were always performed by the same individuals (three surgeons). However, because the groups were divided into two groups according to the disinfection method used during the study period, the ratio of cases performed using laparoscopy increased over time, and a bias existed in the surgical approach between the two groups. Second, the skin of the surgical field was generally disinfected by dipping a sterilized coating material, such as a cotton ball, in sterilized disinfectant and then applying the dipped material to the skin using sterile forceps. PVP-I disinfection was performed using this method. OLG disinfection was instead performed using an applicator in which the disinfectant and the coating material were aseptically integrated. The use of an applicator reduces the burden on medical workers during disinfection procedures, and it may also reduce the risk of bacterial contamination and contribute to the reduction of SSIs because it is sterilized and packaged. For a precise comparison of the efficacy of the disinfectant itself, it may be necessary to perform disinfection using a similar approach in both groups. Finally, several evidence-based guidelines for the prevention of SSIs were updated during the study period; these included antisepsis for preoperative surgical skin preparation according to the World Health Organization (WHO) and CDC and included chlorhexidine-alcohol-based (CHG-AL) agents but not aqueous PVP-I [15, 31]. Furthermore, one trial in which CHG-AL was demonstrated to be superior to PVP-I for preoperative topical antisepsis in clean-contaminated surgery was followed by a meta-analysis and systematic review that confirmed this result [8, 32, 33]. Therefore, further randomized studies aimed at comparing OLG with an alcohol-based agent such as CHG-AL, are needed to verify the effectiveness of OLG.

In conclusion, in this retrospective study, we demonstrated that OLG was more effective than PVP-I for preventing SSIs during gastrointestinal cancer surgery. In particular, the use of OLG may also be more effective in surgeries with a high risk of SSI, such as colorectal cancer. This result indicates that OLG may be useful in reducing SSI in patients undergoing gastrointestinal surgery.

## Data Availability

The datasets used during the current study available from the corresponding author on reasonable request.

## Abbreviations

SSI: Surgical site infection
PVP-I: Povidone-iodine
CHG: Chlorhexidine gluconate
OLG: Olanexidine gluconate
CDC: Centers for Disease Control and Prevention
MRSA: Methicillin-resistant *Staphylococcus aureus*
VRE: Vancomycin-resistant enterococci
